# *Sideroxylon Obtusifolium* (Roem. & Schult.) TD Penn: Knowledge and Potentialities

**DOI:** 10.1007/s11130-026-01475-3

**Published:** 2026-06-22

**Authors:** Luísa dos Santos Conceição, Sanket Prakash Vanare, Ronald Bruce Pegg, Deborah Murowaniecki Otero

**Affiliations:** 1 Graduate Program in Food Science, Faculty of Pharmacy, Federal University of Bahia, Campus Ondina, Salvador, Bahia, 40170115 Brazil; 2 Department of Food Science & Technology, University of Georgia, Athens, GA 30602 United States of America; 3 Graduate Program in Food, Nutrition, and Health, Nutrition School, Federal University of Bahia, Campus Canela, Salvador, Bahia, 40110907 Brazil

**Keywords:** Quixaba, Botanical characteristics, Biological activity, Phenolic compounds, Review

## Abstract

*Sideroxylon obtusifolium *(Roem.& Schult.) T.D. Penn, also popularly known as ‘quixabeira’, is a medicinal species characterized by thorny deciduous vegetation, a short trunk, whitish flowers with a sweet aroma, glabrous leaves, and succulent fruits with seeds. The objectives of this review were to compile and evaluate the literature on quixabeira published over the last fifteen years and to explore the potential applications of each part of the tree. The results of these studies are promising. Studies have shown that the chemical composition of *S. obtusifolium* extracts varies depending on the plant part and the solvent used, with maceration and immersion being the most common techniques, especially for leaves. Methanol and ethanol are the most commonly used solvents to identify compounds such as phenolics, saponins, terpenoids, alkaloids, and glycolipids. The biological activity of the polyphenols present in the leaf extracts demonstrated antioxidant, anti-inflammatory, antibacterial, antifungal, antinociceptive, and antimicrobial properties *in vitro*. In the fruits, extraction is still limited. However, it allows for the identification of carotenoids (47.21 μg/g of β-carotene), flavonoids (55.99 mg of quercetin/100 g), and anthocyanins (58.68 mg/100 g), all of which have nutritional and technological potential. Ethnopharmacological studies indicate that the species is recognized by rural communities, which utilize various parts of the plant for food, medicinal, veterinary, technological, and construction purposes. However, further studies on physicochemical, nutritional, toxicological, and pharmacological composition are needed to ensure safety and validate its biological properties.

**Supplementary Information:**

The online version contains supplementary material available at https://doi.org/10.1007/s11130-026-01475-3.

## Introduction

Fruits and vegetables are essential to global agrobiodiversity. As of today, more than 1,000 vegetable species have been recognized worldwide, and at least 1,250 fruit species have been identified in Latin America alone [1]. This rich diversity of plant species has attracted worldwide attention, contributing to greater interest in studies on these natural resources [2]. In this context, knowledge about the use of native plants is crucial for preserving biodiversity, as they offer potential health, environmental, and economic benefits [3].

The growing research and consumer interest in healthy, sustainable foods have prompted a deep dive into novel food sources [4]. The consumption of Unconventional Food Plants (UFPs) has emerged as a strong candidate for this purpose [5] due to their potential health benefits arising from the abundance of nutrients and bioactive compounds with medicinal properties [4]. UFPs have numerous untapped applications in the food sector, primarily in developing functional foods, warranting studies to evaluate and verify their safety and nutritional benefits [6].

The Sapotaceae family consists of native plants found in Central and South America [7]. These plants have been reported to contain a variety of carbohydrates, fatty acids, and plant bioactive compounds [8]. Within this family, *Sideroxylon obtusifolium* (Roem. & Schult.) T.D. Penn [9], more commonly known as “quixabeira”, stands out as highly relevant for sustainability and environmental preservation [10]. Quixabeira is a tree with a wide geographic distribution, spanning parts of Mexico, Central America, and South America [11]. It is particularly found in dry soil regions extending from Mexico to Argentina [12]. Sightings have also been reported in Venezuela, Bolivia, Paraguay, Uruguay, Costa Rica, and Belize [13].

*S. obtusifolium* is one of the species with great local relevance due to its therapeutic uses [10], specifically the pharmacological properties of the leaves and the bark, demonstrating antibacterial and anti-inflammatory properties [14–16]. Additionally, its fruits are used as food, and the wood is utilized for construction [18]. It also has potential applications as a natural pigment in the food industry [9]. Unfortunately, *S. obtusifolium* is classified as one of the species at risk of extinction in northeastern Brazil, due to its continuous exploitation for bark [9, 18]. This scenario highlights the importance and urgency of further research into its sustainable use and conservation.

Therefore, the objectives of this review were to compile and evaluate the scientific literature published over the last fifteen years, as well as the potential applications of each part of the quixabeira tree. This time frame enables the systematization and updating of scientific knowledge produced during a period marked by significant methodological, technological, and conceptual advances in the area of study.

## Procedure and Data Analysis

 The Procedure and Data Analysis section is described in Supplementary Material.

## Results and Discussion

### Botanical Characteristics of the Tree

**Fig. 1:**
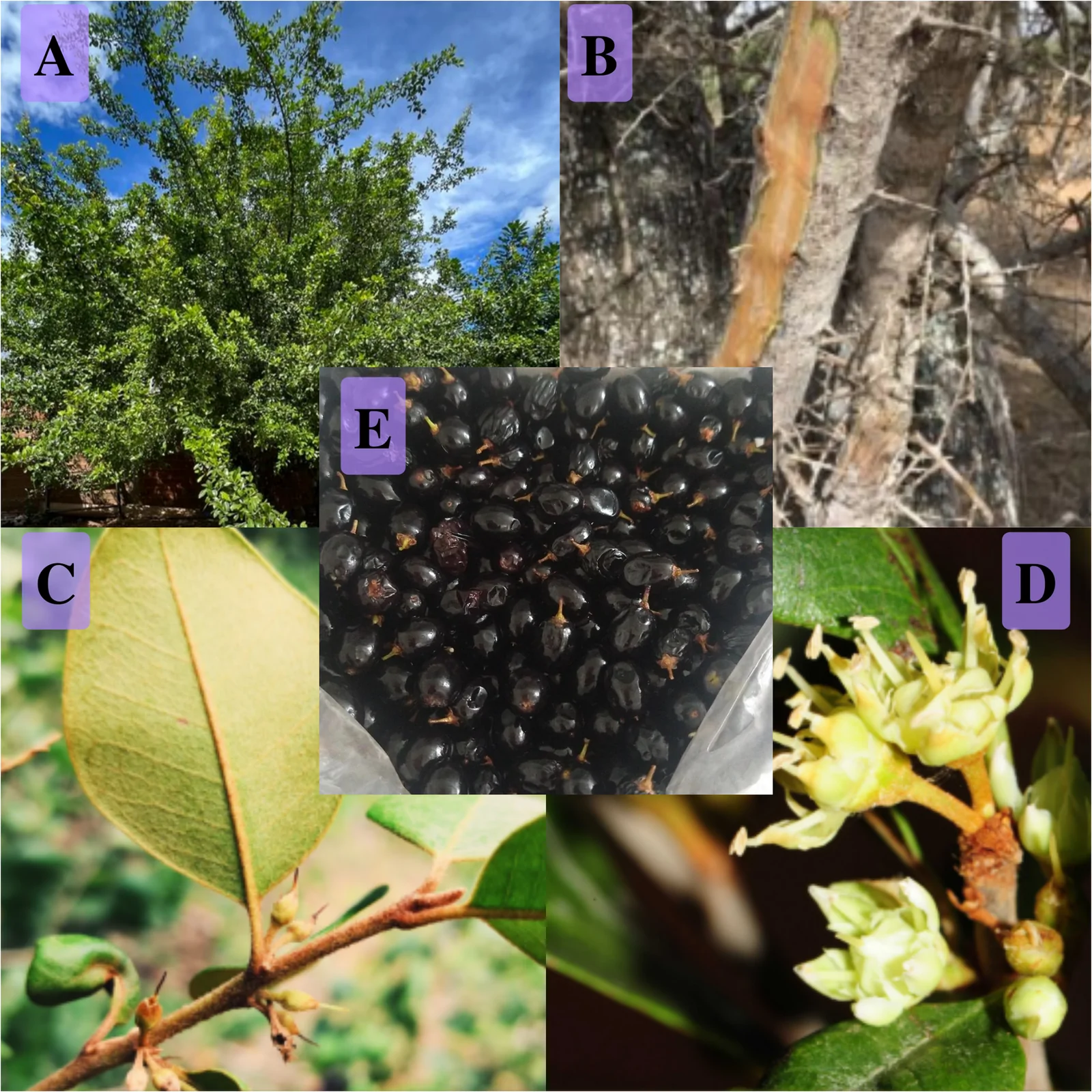
Different parts of the quixabeira: A = tree, B = trunk [24], C = leaf [24], D = inflorescence [25], E = fruit (figure was created with canva.com)

 Quixabeira (Fig. 1) is a medicinal tree species from the Sapotaceae family, native to the Caatinga biome [16]. It is part of the thorny deciduous vegetation, characterized by a dense canopy (Fig. 1A) [201], and can reach heights of 7-18 m [8, 21]. The tree (Fig. 1B) has a short trunk with a diameter of 30-60 cm and a wood density of 0.93 g/cm³, exhibiting surface cracks [20].

*S. obtusifolium* (Roem. & Schult. T.D. Penn) is known popularly by several names, such as "quixaba" [22],"quixaba-preta" [17],"rompe-gibão", "maçaranduba-da-praia","sapotiaba" [22],"sacutiaba", and "miri" [11]. It is also associated with scientific synonymy: *Bumelia sartorum* Mart.;* Bumelia obtusifolium* Roem. & Schut.; *Bumelia rotundifolia* Schut. [23].

*S. obtusifolium* has a reddish-brown stem, branches, and thorns 1.5-3 cm long [13]. The leaves (Fig. 1C) are of a chartaceous type, glabrous and with a shiny surface [22], 1.5-6.5 cm in length and 0.5-3.5 cm in width [13]. Quixabeira is characterized as facultatively deciduous [12], with the possibility of increased leaf production due to the association with riparian vegetation. 

 The flowering phenophase (Fig. 1D) occurs between October and November, while fruiting and ripening occur from January to February [21]. However, the specific climatic conditions of each environment can influence these phenological phases and alter the timelines [12]. A phenological evaluation study of the fruits of zoochoric plants conducted by Silva et al. [26], including *S. obtusifolium*, showed that more significant rainfall favored fruiting from December to March, with a peak in February. 

 Despite the comprehensive morphological descriptions available on the flowers and pollinators of *S. obtusifolium*, the current literature lacks studies on addressing the chemical characterization of floral metabolites. Although the pleasant aroma of the flowers is mentioned, no studies in the current literature have investigated the chemical constituents responsible for this fragrance, nor have any characterized volatile compounds from other parts of the tree, such as fruits and bark. Over the last fifteen years, to date, only one study has evaluated the secondary metabolites of *S. obtusifolium* leaves [19], and another investigated the volatile products formed by the pyrolysis of dehydrated leaves [33], which does not reflect the species natural volatile profile. Thus, future investigations should prioritize the characterization of volatile compounds from both the flowers and other parts of *S. obtusifolium*, expanding the available chemical knowledge and enabling the identification of potential applications.

### Characteristics of the Fruit

The fruits of *S. obtusifolium* (Fig. 1E) are edible and characterized as drupe, indehiscent, monospermic, with a variable morphological shape (globose or ellipsoid), and a smooth and thin skin of a bright purple color. During the collection process, latex exudate can be noticed when the peduncle is detached from the plant [22, 27]. 

The fruit pulp is juicy, gelatinous, and sweet [28], and the ripening process is noticeable by the change in color of the skin from dark green to dark purple [23]. The morphological characterization of quixabeira fruits shows average length, weight, and diameter of 12.12 mm, 1.06 g, and 10.41 mm, respectively [22]. In another study, the reported average fruit weights, diameters, thicknesses, and lengths were 1 g, 10 mm, 0.5-2 mm, and 12 mm, respectively [27].

### Seeds

The fruiting of Caatinga trees, such as *S. obtusifolium* and *Z. joazeiro*, occurs only during the rainy season, resulting in a large production of fleshy fruits. These fruits are consumed by frugivorous wild animals [26], contributing to the zoochorous seed dispersal [28] and ecological relevance as a food source [27]. 

Water is an essential resource for the fruiting process of Sapotaceae family trees [12], in addition to other environmental factors, such as sunlight, soil, and topography [26]. The specific regional climatic conditions of each biome influence the variation in the phenophases of flowering and fruiting of the tree [29]. 

The tree is propagated by seed, with approximately 2000 seeds *per* kg of fruit [20]. The seeds of *S. obtusifolium *evaluated in the study by Silva et al. [22] were characterized as bitegumented, varying from globose to ellipsoid in appearance, and light brown. They had an average mass of 0.15 g, length of 8.85 mm, and diameter of 5.40 mm. The seeds are classified as neutral photoblastic and have a greater possibility of germination at temperatures between 25-30 °C [22].

*S. obtusifolium* seeds are physically dormant and need to undergo physical or chemical treatments to become viable for germination [27]. Seeds of *S. obtusifolium* were subjected to pre-germination treatments of chemical (with sulfuric acid), mechanical (using sandpaper), and thermal scarification. The author highlights that this tree species exhibits hypogeal germination, in which the cotyledons remain in the soil, and that the emergence of the epicotyl serves as an indicator of successful germination. Scarification using sulfuric acid for 20-30 minutes was the method identified in the study, favoring greater germination speed and initial growth of the species [28]. Furthermore, it is essential to carry out hygiene procedures before sowing to prevent fungal infestation. Washing with running water, alcohol (70%), and sodium hypochlorite (1%) is recommended. 

The physiological maturity of *S. obtusifolium* seeds (n = 30), evaluated by Sena et al. [21] in a rural area, occurred 105 and 112 days after the anthesis phase, demonstrating high physiological quality. This stage was characterized by a reduction in water content (23.6% at 112 days after anthesis) and the accumulation of maximum dry mass (1 g at 84 days after anthesis).

### Characteristics of the Flowers and Floral Visitors

Inflorescences initially emerge in the peripheral axillary fascicles and then in the central part [12]. Specifically, 5 to 25 flowers appear between the stem and the leaves, with a diameter of 5 to 6.4 mm and a length of 6.9 mm [29] (Fig. 1D). The flowers are classified as pentamerous, hermaphrodite [12], diurnal, protogynous, nectariferous, actinomorphic, and herkogamous [29]. They are whitish and stand out for their pleasant, sweet aroma [30]. 

The morphological characterization of quixabeira flowers, as described in the literature, reveals that the corolla is gamopetalous and pentamerous, measuring approximately 1.5 mm in depth [29]. The pollen release apparatus is located in the stamen [12], where it is possible to identify three lacinia (two lateral and one median), 5 mm long [29]. At the center of the trends is the androecium, which consists of five stamens and staminodes [12]. The latter are classified as petaloid, with a lanceolate appearance and a length of 3.4 mm [29]. The fillets can be cream-colored [12] or white with 3.9 mm [29]. 

The anthers are characterized as pointed and extruded, with a rhymed opening and yellow color [29], but they turn light brown as they mature [12]. The stigma is classified as papillose, lobulated, and with a moist appearance, measuring 0.41 mm in diameter. The style and pollen grains are white, measuring 5.3 mm and 26 μm, respectively [29]. The nectary has a ring shape surrounding the upper ovary (1-1.3 mm in diameter and 1.7 mm in length) where the gynoecium is located, characterized as pentaovulate and with a single style [12]. 

Like other species in the *Sapotaceae* family, the quixabeira produces a small amount of nectar in the form of droplets, which makes quantification impossible [29]. It is an important factor in encouraging the increased movement of pollinating insects between flowers, thereby facilitating the dispersal of pollen grains [12]. 

The floral visitors of *S. obtusifolium* (Fig. S2) include the orders Hymenoptera, Lepidoptera, Coleoptera, Diptera, and Thysanoptera. These are more frequent in the hermaphrodite phase due to better access to nectar and greater appeal to the appearance and smell of the flower [29]. The bee species *A. mellifera* and dipteran morphospecies (1 and 2) were identified as pollinating agents in the study conducted by Kiill et al. [12]. When accessing the stigma of the flower to collect nectar, they ended up with a body entirely covered in pollen grains. 

The quixabeira's other floral visitors include the butterfly *Isanthrene incendiaria* and Coleoptera(beetles), such as *Astylus lineatus* and *Oedemeridae*, which are classified as nectar robbers because they do not touch the stigma of the flower. Kiill et al. [12] identified 17 species of insects visiting the flowers of *S. obtusifolium*, including Hymenoptera (n = 8), Diptera (n = 8), and Lepidoptera (n = 1). 

Gomes et al. [29] reported ants, Diptera, and Thysanoptera insects as nectar gatherers. At the same time, the pollinators were beetles (*Astylus linneatus* and *Oedemeridae*), butterflies (*Isanthrene incendiaria*), and bees (*Apis mellifera, Xylocopa ordinaria*, and *Brachygastra lecheguana*). The bee (*Trigona spinipes*) was observed both as a nectar gatherer and as a pollinator. 

In addition to insects interested in quixabeira’s daytime flowers, the fruits were identified by the presence of fly larvae by Coelho and Uchoa [31]. Fruits (976, total weight of 1.5 kg) of *S. obtusifolium*, classified as a common species of Wooded Steppic Savanna, demonstrated the presence of 19 larvae of the fly of the genus *A. obliqua* (7 adults, four females, and three males). Samples of quixabeira and other fruit trees from the Brazilian Chaco region were used in this study, which is a dry subtropical forest vegetation region also found in Argentina, Paraguay and Bolivia. 

The presence of vectors was identified as an essential factor for fruiting quixabeira, as reported by Kiill et al. [12], which shows that cross-pollination favors more significant fruit formation relative to the number of flowers compared to natural conditions.

### Bioactive Compounds and Biological Potentialities

 Phytochemicals, or bioactive compounds, are plant-derived metabolites that act as a defense mechanism against environmental threats [33]. Milanezzi [34] highlights the growing interest in fruits and vegetables rich in bioactive compounds, due to the nutritional benefits associated with their antioxidant activity. 

 The literature indicates a diversity of phytochemicals present in *S. obtusifolium* (Table S1). In the leaves, tannins [30], flavonoids, catechins, phenols, alkaloids [15], saponins [14], monoterpenes, sesquiterpenes, triterpenes, condensed proanthocyanidins and leucoanthocyanidins [35], can be found. The bark has been reported to contain polyphenolic compounds [15], and the fruit has been found to contain carotenoids, flavonols, and anthocyanins [36]. Studies indicate the use of organic solvents (ethyl acetate, methanol, and acetone) as the primary extraction method for the compounds, which are subsequently identified by analytical techniques, including mass spectrometry and column chromatography. The chemical composition of quixabeira varies according to the plant’s development phases, necessitating more studies to understand these metabolic changes throughout the cycle, as factors such as seasonality, phenological aspects, ecological conditions, and genetic variations also influence these variations [30]. 

Phenolic compounds are characterized chemically by an aromatic ring with one or more hydroxyl groups [37]. They are present in different parts of plants, standing out for their antioxidant properties and the potential to affect the nutritional and sensorial quality (color, texture, and flavor) [37]. Phenolic compounds include phenolic acids, flavonoids, lignins, coumarins, and tannins [33]. Notable bioactive compounds identified in *S. obtusifolium* include flavonoids (anthocyanins), carotenoids, and condensed tannins. The health benefits associated with flavonoids, including their anti-inflammatory effects and the reduction of LDL cholesterol, have garnered significant interest in this group of bioactive compounds [34]. 

Do Nascimento et al. [36] characterized the proximate composition and quantification of anthocyanins, flavonols, and carotenoids in Caatinga trees, including *S. obtusifolium, *which reported 55.99 mg quercetin/100 g of fruit for flavonols, 47.21 β-carotene μg/g for carotenoids, and 58.68 mg anthocyanins/100 g. The proximate analysis revealed 57.39 g of moisture, 1.23 g of ash, 28.50 g of carbohydrates, 2.86 g of protein, and 9.62 g of lipids per 100 g of fresh fruit weight. 

The carbohydrate content of the quixabeira fruit (28.50 g/100 g fruit) was the highest when compared to the other 11 trees [36], surpassing *Psidium schenckianum* (26.60 g/100 g fruit) and *Ziziphus joazeiro* (19.38 g/100 g fruit). It was ranked second in lipid content, only behind *Syagrus cearensis* with 69.33 g/100 g fruit. The total caloric value in the fruit was 212 Kcal/100 g, the second-highest value after *S. cearensis*, with 392 Kcal/100 g. The daily caloric needs of an adult and a child are met by 6% and 10%, respectively, with the consumption of 100 g of fruit. 

Anthocyanins are natural pigments that have antioxidant, anti-inflammatory, and cardioprotective actions [33]. The color (violet, blue, magenta, or red) of various plant parts, such as fruits and flowers, is a result of the presence of anthocyanins [38]. Anthocyanin was found both in the pulp (30.49 mg cyanidin-3-*O*-glucoside/100 g fresh weight) and in the peel (236.15 mg cyanidin-3-*O*-glucoside/100g fresh weight) of *S. obtusifolium *fruits, as reported by Figueredo and Lima [23]. Similarly, Do Nascimento et al. [36] reported an anthocyanin content of 58.68 mg/100 g in the fruit. 

 The anthocyanin content found in the peel of quixabeira fruits is higher when compared to other purple fruits considered rich in this pigment, such as 6.7 - 154 mg/100 g in grapes [39], 73.54 mg/100 g in açaí [40], and 58.95 mg/100 g in blueberry pulp [37]. It is worth highlighting that grapes are fruits rich in phenolic compounds, emphasizing the significant influence of anthocyanins on color and other critical sensory attributes for beverage production [39]. Anthocyanin is also one of the polyphenols that make up the açaí pulp and is responsible for the antioxidant activity of this fruit [40].

Studies on the application of natural pigments in processed foods as an alternative to synthetic dyes have increased, as has consumer demand for clean-label products [38]. The biological properties of pigments of natural origin, along with their perceived sustainability compared to synthetic alternatives, have attracted the food industry's interest in research aimed at developing biocolors [41]. Environmental factors, such as luminosity, temperature, and pH, are among the factors that affect the stability of anthocyanins, posing a challenge to their applications in industrialized food products [38]. To address this, Koop et al. [42] encourage studies on encapsulation and adsorption technologies as means of improving and analyzing anthocyanin stabilization. 

Tannins are another group of phytochemicals present in *S. obtusifolium*, identified as the primary influencers on its medicinal properties [30]. The classification of tannins depends on their chemical structure; they can be classified as condensed (*i.e.*, proanthocyanidins) or hydrolyzable [37]. Gomes et al. [30] quantified the concentration of tannins present in the aerial part and periderm of the stem of *S. obtusifolium *(n= 6) in different granulometric ranges. The highest tannin concentrations reported were 30.45 mg/g and 51.36 mg/g for periderm (particle size 149-74 μm) and aerial part (particle size <74-37 μm), respectively. The aerial part demonstrated a higher tannin content compared to the periderm of the stem, which is the most popularly used part. The size of the particles and the environmental variability of the species might have influenced the results. 

Studies on *S. obtusifolium* (Table S1) demonstrate that the extracts have variable chemical composition, depending on the tree part, with maceration or immersion of the sample in solvent being the two most commonly used extraction strategies. In some cases, extraction can also be performed using the Soxhlet method [8]. Leaves are the most frequently reported part in the evaluated studies. Among the solvents identified, methanol is used in the production of extracts in studies evaluating proline derivatives (NMP), highlighting the diversity of approaches used to isolate bioactive metabolites [7, 15, 35, 43, 44]. Other solvents are also used to obtain leaf extracts, such as hydroalcoholic solution [16] and ethanol [8], the latter being associated with subsequent sequential partitioning with other solvents (hexane, dichloromethane, ethyl acetate, and butanol) to generate extract fractions. Both are used to evaluate flavonoids in the samples. In the case of the tree bark [14, 45], ethyl acetate allows the identification of polyphenolic compounds. At the same time, ethanol is used for both the bark and the inner bark [46], enabling the detection of phenolics and terpenes. 

Despite the smaller number of articles found, solvents such as acetone, hydrochloric acid, ethanol, and chloroform are indicated as the most used for the production of extracts using the fruits, associated with the identification of anthocyanins, carotenoids, and flavonoids. Although these analyses with extracts enable the identification of metabolites with potential applications in various sectors, primarily in the pharmaceutical field, further research using different methodologies (such as HPLC and GC-MS) is still necessary to elucidate and quantify the chemical components originating from different parts of the tree. 

 The efficiency of the extraction process is determined by a series of interrelated factors, including the physicochemical properties of the solvent, the particle size of the raw material, the solvent-to-solid ratio, temperature, and the contact time between phases. Among these elements, solvent selection plays a central role, as its nature defines the ability to solubilize the target compounds and, consequently, the yield and purity of the extract obtained. Therefore, criteria such as selectivity, solubility, operational cost, toxicity, and handling safety must be carefully evaluated to ensure an efficient and sustainable process that is well-suited to the characteristics of the matrix being extracted. Therefore, future studies should focus on characterizing the bioactive compounds, especially those found in the fruits, thereby deepening scientific knowledge about the species and enabling better targeting of its potential applications in the food sector.

### Biological Activity

The bioactive compounds present in *S. obtusifolium* demonstrate therapeutic potential (Fig. S3) in the form of antibacterial, antioxidant [14], anti-inflammatory [33], wound-healing [10], antifungal [10, 17], antimicrobial [16], antinociceptive [7], and antiandrogenic properties [14]. Figueiredo and Lima [23] indicated the medicinal potential of extracts obtained from quixabeira leaves and stems, as they are applied to treat wounds and inflammations. 

The leaves, bark, and pieces of the trunk in dry form [10] of quixabeira are most recommended for obtaining extracts for medicinal purposes, mainly to combat inflammation [23]. Pereira et al. [16] highlighted the importance of evaluating the antimicrobial properties present in other parts of quixabeira. 

Aquino et al. [7, 35, 43, 44] investigated the biological effects of *S. obtusifolium *leaf extract, enhanced by the presence of bioactive compound*N*-methyl-(2*S*,4*R*)-*trans*-4-hydroxy-L-proline (NMP). The chemical compound NMP, extracted from the leaves, demonstrated antinociceptive and anti-inflammatory properties in mouse models, as well as reducing myeloperoxidase activity *in vitro* assays with human neutrophils [7]. The authors evaluated the antinociceptive effect of NMP in mice using the formalin test. The results demonstrated that all NMP doses tested (25, 50, and 100 mg/kg) reduced nociceptive behavior in both phases of the test. The first phase, related to direct chemical stimulation of nociceptors, showed reductions in licking time of 35%, 42%, and 52%, respectively. The second phase, associated with peripheral inflammatory processes, showed reductions in licking of 30%, 61%, and 78%, respectively. The authors also used different concentrations of NMP (25, 50, and 100 mg/kg) to evaluate its anti-inflammatory effects in a carrageenan-induced paw edema model. In this context, treatment with NMP decreased paw edema volume by 30, 37, and 51%, respectively. Furthermore, in the carrageenan-induced peritonitis model, administration of NMP (100 mg/kg) reduced polymorphonuclear cell infiltration by 77%. Additionally, results from an *in vitro* assay demonstrated that NMP (25, 50, and 100 μg/mL) reduced myeloperoxidase activity in human neutrophils, decreasing absorbance by 24, 31, and 40%, respectively. Based on these results, the authors suggest that NMP has promising potential for the development of new analgesic and anti-inflammatory drugs. 

 The compound was incorporated into a NMP topical gel (3% and 10%), which demonstrated anti-inflammatory action and promoted wound healing in rats [35]. Histological, biochemical, and histochemical analyses showed that the gel stimulated extracellular matrix proliferation and decreased protein carbonylation. The authors indicate that treatment with 10% NMP resulted in a decrease in wound area, reduced microscopic changes, and increased collagen deposition, observed on day 7 (approximately 45%) and day 12 (approximately 58%). Furthermore, the results demonstrated that this concentration of NMP improved the healing process, evidenced by a reduction in lipid peroxidation (approximately 41%) and myeloperoxidase activity (52% reduction), as well as an increase in inducible nitric oxide synthase and cyclooxygenase-2 activity (both around 26%) and an elevation in glutathione levels (2.3 times), when compared to the control group. Aquino et al. [35] attribute the tissue repair effect to the presence of L-proline, since the NMP present in the leaves of *S. obtusifolium *is derived from this amino acid, which, along with hydroxyproline and glycine, participates in the formation of collagen, a fundamental structure for the cicatrization process. 

The potential neuroprotective action of NMP extract has been reported by Aquino et al. [43] in their evaluation of the intracerebroventricular pilocarpine model. The concentration of NMP was determined by quantitative proton nuclear magnetic resonance, yielding 121.7 ± 5.1 mg/g in the methanolic fraction of the leaves. The authors emphasize that oral treatment of mice with different concentrations of NMP (100 mg/kg and 200 mg/kg) was able to prevent short-term memory impairment associated with pilocarpine-induced lesions, since the performance of the treated animals did not differ from that of the control group. In addition, the results of the Y-maze test demonstrated that NMP treatment at both doses prevented cognitive deficits, indicating preservation of the mice's memory function [43]. Another study also used NMP to investigate the anticonvulsant effect in mice, specifically in astrocytes exposed to cytotoxic concentrations of pilocarpine [44]. The authors report that treatment with NMP (25 mg/mL) protected astrocytes against pilocarpine-induced lesions in a dose-dependent manner. Furthermore, NMP significantly reduced the accumulation of cytoplasmic reactive oxygen species in the groups treated with 25, 50, and 100 mg/mL, with results of 27.3, 24.8, and 12.3%, respectively. The results showed a potential neuroprotective effect of high doses of NMP extract, which prevented the onset of cognitive deficits and cellular damage. They increased the expression of GFAP, Iba-1, and GAT1 in the hippocampus. The reduction in ROS accumulation, improvement in mitochondrial potential, and overexpression of VDAC-1 (a regulator of mitochondrial function) are proposed as possible mechanisms underlying the anticonvulsant and neuroprotective actions of NMP in astrocytes [44]. However, more research is needed on the isolated action of these compounds present in the extract. 

Studies have also been conducted to investigate and identify phytochemicals in the leaves of *S. obtusifolium*. Chemical characterization of the methanolic extract reported by Aquino et al. [15] revealed the presence of polyphenols (150.3 mg gallic acid equivalents/g) and flavonoids (98.5 mg quercetin equivalents/g), suggesting that these biological effects may be related to the presence of phytochemicals recognized for their pharmacological properties. However, the authors emphasize the need for further studies to elucidate the mechanism of action of the antioxidant activity of the isolated compounds and to define their specific properties [15]. The phytochemical profile of the leaves, described by Oliveira et al. [19], identified 16 compounds, including four triterpenic saponins, ten flavonol glycosides, as well as catechin and the glycerogalactolipid gingerglycolipid A. Characterization was performed by HPLC-PDA-MS, complementary chromatographic techniques, and spectroscopic analyses. The ethanolic extract of the leaves (149 g) was obtained by exhaustive extraction with ethanol, followed by evaporation under reduced pressure, and subsequently subjected to successive partitions. The authors indicate that among the identified compounds, three were described for the first time: a new triterpenic glycoside and two new flavonol glycosides derived from quercetin and kaempferol [19]. 

The anti-inflammatory activity and possible antioxidant action originating from *S. obtusifolium *were also highlighted by Leite et al. [46] when they applied the ethanolic extract of the bark in a skin healing model studied in rats. The authors indicate that the ethanolic extract (3, 10, and 30 mg) was incorporated into 0.4 mL of moisturizing cream for subsequent topical application in rats. Among the doses evaluated, treatment with the highest amount of extract (30 mg 0.4 mL^-1^) administered twice daily significantly impaired myeloperoxidase activity and cell count in sponge exudate 48 hours after wound induction. As indicated by previous studies from the research group itself, the authors suggest that the anti-inflammatory activity of the extract is related to the presence of phytochemicals (tannins and flavonoids), which have demonstrated the ability to reduce leukocyte infiltration [46]. In addition to the application in rat tissues, the anti-inflammatory action of quixabeira leaf extract was also evaluated in human cell wounds [10]. The effect of *S. obtusifolium* leaf extract on cell proliferation and migration was evaluated using an *in vitro* wound healing test, using different concentrations (25, 50, and 100 µg/mL). After 24 hours of treatment, there was a reduction in the open wound area of 14.5% and 34% at concentrations of 25 µg/mL and 50 mg/mL of extract, respectively, compared to the control. This effect persisted for 48 hours, with reductions of 28% and 38% at the same concentrations. At the end of 72 hours, the open area showed even more significant reductions, reaching 42.1% (25 µg/mL) and 72.6% (50 µg/mL) of the extract compared to the control. The results indicated that the methanol fraction of the extract stimulated human keratinocytes and promoted healing by modulating inflammatory mediators in mice with burn injuries. 

The antiandrogenic effect was identified in a study conducted by Bobach et al. [14], which utilized an ethyl acetate extract (25 mg/ml) obtained from the bark of *S. obtusifolium* and demonstrated an inhibitory effect (-56%± 14%) on LNCaP cell proliferation mediated by testosterone. The antiandrogenic effect was inferred from the extract's chemical profile, as thin-layer chromatography revealed the presence of polyphenolic compounds (epicatechin, catechin, and proanthocyanidins) [14]. However, the authors only suggest a possible association between these phenolic compounds and the observed activity; further studies are needed to elucidate the molecular interactions involved, the biological effects, and to consistently establish the relationship between the identified metabolites and the antiandrogenic effect. These results demonstrated a potential application in the cosmetic industry for acne, hirsutism, and androgenic alopecia. 

The butanol-soluble fraction of *S. obtusifolium* leaves and a nanoemulsion made with the extract were studied by Rangel et al. [8] to investigate their antiparasitic effect when applied to adult and young species of *Biomphalaria glabrata*. The SOB-extract (butanol fraction) and nano-SOB (nanoemulsion) showed interesting results in controlling the adult population, with lethal concentrations of LC^50^: 125.4 mg/L and LC^90^: 178.1 mg/L, and an LC^50^ of 75.2 mg/L and an LC^90^ of 97 mg/L, respectively. The nano-SOB formulation showed greater potency in the young population of the parasite, with an LC^90^ of 72.1 mg/L and an LC^50^ of 58.3 mg/L. It also reduced viable spawning by approximately 30% at 178.1 and 97 mg/L concentrations, demonstrating the potential to inhibit *B. glabrata* growth and disease transmission. Quercetin-3-rhamnosyl-(1-6)-galactoside and hyperoside were two compounds in the extract that stood out in *in silico* analyses for their biodegradability, low human toxicity, and potential for environmental accumulation. Hyperoside is a chemical compound found in plants that exerts several beneficial health effects, most notably its antioxidant, anti-inflammatory, and antifungal properties [47]. 

Duarte et al. [17] investigated the potential fungicidal activity of a quixabeira leaf extract through *in vitro* antimicrobial assays. The ethyl acetate fraction obtained from the *S. obtusifolium* leaf extract exhibited a fungicidal activity of 12.5 mg/mL, suggesting it as a possible tool to help control *T. ethacetica *fungus infestations in food crops [17]. Sampaio et al. [45] reported evaluating the *in vitro* antimicrobial activity of bioactive extracts using both the quixabeira leaf and bark on *Streptococcus* (*mutans*,*oralis*,*salivarius*, and *parasanguinis*) and *Candida albicans*. Although all extracts (lyophilized hydroalcoholic and ethanolic) of the leaf and bark evaluated in the study showed moderate inhibition of *C. albicans* growth (Minimum inhibitory concentration = 500 μg/mL), the dichloromethane and n-butanol fractions of the leaf extract demonstrated significant antifungal potential (Minimum fungicidal concentration/Minimum inhibitory concentration = 2). The authors indicate that the reported fungicidal action may be related to the phytochemicals (condensed tannins and polyphenols) present in the analyzed extracts, but further studies are needed to determine whether the extracts have a fungistatic or fungicidal effect. 

Although several studies indicate that *S. obtusifolium* extract has therapeutic potential, mainly when obtained from the leaves and bark, few offer detailed explanations of the molecular mechanisms underlying the reported biological effects. Therefore, studies are needed to elucidate these mechanisms, particularly about their biological properties, as well as investigations that include other parts of the plant, such as the fruits and flowers, which are still underexplored in the current scientific literature.

### Ethnopharmacological Knowledge

The traditional knowledge of communities about the medicinal properties of native plants can significantly contribute to scientific research [48]. Ethnopharmacological studies of native plants help identify properties for health applications and safe use. 

*S. obtusifolium *was one of the 56 plant species identified in the study by Barbosa et al. [2], conducted between 2011 and 2012, to evaluate the interrelationship between residents of a rural community and environmental information on useful woody plants. Quixabeira was included in the group of native species highlighted in phytosociological research. The bark, inner bark, leaf, fruit, and wood were noted to be the most used parts. Regarding the applications indicated by popular knowledge, the answers included food, fuel, construction, forage, medicine, technology, veterinary, and others [2]. 

The bark of the stem and inner bark were the parts used by the traditional communities mentioned in the ethnobiological study carried out by Ribeiro et al. [48]. *S. obtusifolium* was one of nine among 61 medicinal species identified with the greatest need for conservation and traditional medicinal practices for more sustainable use. The quixabeira was among the 82 plants mentioned in the study by Da Mata et al. [49], who evaluated the knowledge of young people from a public school about Caatinga trees. 

The bark and inner bark obtained from the youngest *S. obtusifolium* trees are the parts most commonly used by traditional communities due to the benefits they offer against diseases such as duodenal ulcers, gastritis, diabetes, kidney, genital, and heart problems, among others [30]. These effects are possibly associated with the presence of polyphenols and other metabolites identified in the studies included in this review (Fig. S3), considering the previously reported antibacterial [14], antifungal [45], and antioxidant [14, 46] activities. Along with the bark, another part highlighted for medicinal use is the leaves, which are used in tea preparations [15]. The trunk is used to build sculptures for crafts or carpentry, the flowers are melliferous, and the fruits can be consumed by humans or used as animal fodder, along with young leaves, showing that all parts of *S. obtusifolium* can be utilized [13]. 

Despite the abundant use of *S. obtusifolium* in folk medicine, this wealth of ethnopharmacological knowledge has been lost over time [15]. The slow growth of the tree and the decrease in vegetation [30] due to its intense use for medicinal and economic purposes [48] may exacerbate these concerns. 

In ethnopharmacology, bioactive compounds derived from medicinal plants are associated with the treatment of pain, inflammation, infections, digestive, and metabolic disorders. In contemporary medicine, these molecules are increasingly investigated as functional biomarkers, nutraceutical ingredients, therapeutic adjuvants, and promising components for drug discovery. Their medicinal activity arises from a wide range of cellular and molecular mechanisms. They neutralize reactive oxygen and nitrogen species through electron or hydrogen donation, chelation of pro-oxidant metals, and induction of endogenous antioxidant defenses, thus mitigating oxidative stress and lipid peroxidation. 

Furthermore, many bioactive compounds exert antimicrobial effects by disrupting microbial membranes, inhibiting essential enzymes, and suppressing biofilm formation. Certain bioactive compounds also exhibit anticancer properties, inducing apoptosis, interrupting the cell cycle, inhibiting tumor angiogenesis, and modulating proliferative pathways. Collectively, these mechanisms reveal the multifaceted therapeutic potential of bioactive compounds and reinforce their relevance in both traditional medical practices and modern biomedical research.

### Areas of Application

 The biochemical activity arising from different parts of the *S. obtusifolium* tree has demonstrated potential for developing pharmaceuticals [16] and herbal medicines [8], owing to their healing [30], antifungal, antimicrobial, antibacterial [45], anti-inflammatory, and antidiabetic [28] properties, as reported in the literature. 

Despite the potential of some medicinal plants for use in the development of medicines, studies must be conducted to evaluate their effectiveness, quality, and toxicological aspects, thereby avoiding negative impacts on consumer health. Correia et al. [32] characterized the chemical composition of dehydrated quixabeira leaves, evaluated in particles of different sizes, by subjecting them to scanning electron microscopy (SEM), thermoanalytical techniques [thermogravimetry (TG) and differential thermal analysis (DTA)], and pyrolysis coupled to gas chromatography/mass spectrometry. The pyrolysis results were grouped according to temperature variation (250, 350, 450, and 600 °C), with the most significant results identified at temperatures of 350 and 600 °C. These being, at 350°C: pentanal; palmitic acid; 2,6,10,14,18,22-tetracosahexane; 1-fluoro-2,2,4,4-tetramethyl-3-pentanone; methyl pulegenate; and methyl comate. At the temperature of 600 °C, the following compounds were identified: limonene; palmitic acid; 4-hexenoic acid; columbine component; methyl pulegenate; and methyl chromate. 

 Quixabeira also has potential applications in the food industry, but further exploration is needed. The enzyme in the latex obtained from the *S. obtusifolium* trees stood out in the investigation by Silva et al. [50]. This study focused on proteases derived from plants in the Brazilian semiarid region and their respective milk coagulation activities for cheese production. Quixaba latex demonstrated superior action to chymosin, considered the most effective plant enzyme for milk coagulation. The specific coagulation activity was 917 U/mg, and the milk coagulation and proteolytic activity ratio was reported as 25.38 U/mg. 

This tree presents a range of characteristics that the food, pharmaceutical and construction industries can exploit. Natural food pigments/additives due to the presence of anthocyanins [23]; bioenergy using wood [27]; phytotherapeutics and nutraceuticals due to bioactive compounds; beverages (wines and juices), as well as sweets and jams due to the presence of soluble solids in the pulp [36], are explored in the literature. The biochemical composition of the quixabeira fruit and its nutritional aspects can facilitate the development of functional foods and promote their consumption in rural areas [36]. 

Nevertheless, further studies are needed to analyze the physicochemical and nutritional composition of the leaves and fruits of *S. obtusifolium*, to understand their potential in food applications and their impact on human health. Toxicological and pharmacological analyses are also essential to ensure safe use and a deeper understanding of the bioactivity of quixabeira, which is widely used in traditional medicine [13].

### Temporal Evolution

All 35 articles selected for this manuscript were distributed according to the year of publication (Fig. S4), ranging from 1 to 5. In 2022 stood out with the highest number of publications (n = 5), followed by 2020 and 2016, which each had four. In most publication years, two articles were identified. Only one article was reported in 2013 and 2011. No articles were identified in 2018. Among the 35 articles (Table S2), the majority were authored by researchers from Brazil (n = 33), with only two articles published by authors from Switzerland (n = 1) and Germany (n = 1). The only study published in 2011 discussed the proximate analysis and chemical characterization of *S. obtusifolium, *andquantified the anthocyanins and carotenoids in species native to Caatinga. 

In 2012, three articles were published about the quixabeira leaf, fruit, and seed, two in Brazil and one in Switzerland. Silva et al. [22] conducted the morphological characterization (internal and external) of the fruits and seeds and provided descriptions and illustrations of the seedlings. The seeds were also the subject of a study by Rebouças et al. [28], who developed a method to overcome their integumentary dormancy. The metabolic profile of *S. obtusifolium* leaves was investigated by Oliveira et al. [19] and published in Switzerland. 

In the only article published in 2013, Silva et al. [50] evaluated the potential of the enzyme present in the latex obtained from the quixabeira tree for cheese production, thereby demonstrating its potential application in the food industry. 

 In 2014, two publications evaluated the entire tree, and another study focused on the bark (trunk), phenology, and biological activity. Kiill et al. [12] studied the phenology, reproductive biology, and floral visitors of quixaba. On the other hand, Bobach et al. [14] assessed the bark extract for its hormonal activity and cytotoxic effects when applied to prostate cancer cells. 

The two articles identified in 2015 analyzed the antioxidant activity of*S. obtusifolium* using different parts of the tree (fruit and bark). The antioxidant activity of anthocyanins present in ripe fruits was investigated by Figueiredo and Lima [23]. Leite et al. [46] used the ethanolic extract from the quixabeira bark to evaluate the topical antioxidant and anti-inflammatory effects on wounds. 

In 2016, four studies were conducted on the quixabeira leaves and seeds. Correia et al. [32] characterized the medicinal properties of the leaves by different particle sizes to promote their use in herbal medicines. Pereira et al. [16] carried out a chemical characterization of leaves, while Aquino et al. [16] used a leaf extract to study its components and evaluate its antimicrobial effect on *Streptococcus oralis*,*Streptococcus salivarius, Streptococcus parasanguinis*, and *Candida albican*s. Melo et al. [18] studied the antifungal effects of seeds. 

The leaves and barks were the parts of the quixabeira highlighted in the two articles published in 2017. Aquino et al. [7] investigated the anti-inflammatory and antibacterial properties of the methanolic extract of leaves. Sampaio et al. [45] evaluated the *in vitro* antimicrobial activity of NMP obtained from leaf and bark extract. 

Quixabeira's leaves were also the focus of 2 publications in 2019. The biological potential of the NMP fraction, obtained from the leaves, was evaluated by Aquino et al. [35]. The objective was to investigate the topical effects of NMP gel, which has anti-inflammatory and antioxidant actions, in a wound-induced model in rats. Meanwhile, *S. obtusifolium* was one of the 61 woody plants classified by Ribeiro et al. [48] as having a conservation priority. 

The entire tree (leaves, fruit, and seeds) was featured in articles published in 2020. Jacob et al. [51] conducted a systematic review to identify food plants supporting food safety and nutrition. Quixabeira was one of 65 species included in this study. *S. obtusifolium* was also one of the zoochoric plant species described in the study by Silva et al. [26]. Aquino et al. [43] used a fraction enriched with an extract from the leaves of *S. obtusifolium* to examine the effects on behavioral changes and the brains of rats. The local botanical knowledge of farmers in the semiarid region was studied by Barbosa et al. [2] through an ethnobotanical inventory to describe and analyze the relationship between the rural community and plants. Quixabeira was one of 56 woody species identified through the semi-structured questionnaire to identify whether participants recognized it and its applications. 

 The three publications in 2021 addressed the phenology and biological action of the extract made with *S. obtusifolium*. Souza et al. [10] used a methanol fraction extracted from leaves (MFSOL), enriched with a proline derivative, to investigate the effects on human keratinocyte cells and burn-type injuries. Gomes et al. [11] evaluated the relationship between phenological intensity and tannin production in the aerial part and periderm of the stem of *S. obtusifolium*. Another study on phenology was carried out by Gomes et al. [30] to develop and test an analytical model that determines parameters for the sustainable collection and medicinal use of quixabeira. 

In 2022, 5 articles addressed the biological effects and phenology of quixabeira. The leaves were used by Duarte et al. [17], Aquino et al. [44], and Rangel et al. [8] to prepare an extract for further assays. Duarte et al. [17] performed *in vitro* antimicrobial tests with the extracts of *S. obtusifolium* to evaluate their fungicidal potential. Aquino et al. [44] extracted NMP from quixabeira to investigate its effect when applied to pilocarpine-induced injury in astrocytes. Rangel et al. [8] formulated a nanoemulsion with the SOB extract and applied it to the biological cycle of schistosomiasis to evaluate its effectiveness. Quixabeira seeds were studied by Sena et al. [21] to determine their physiological maturity point. The study, which utilized the entire tree, was conducted by Cruz et al. [9], who evaluated the phenophases of *S. obtusifolium* in relation to environmental conditions in the Caatinga region. 

Two publications from 2023 focused on widespread knowledge of the tree and parasites in the fruit. *S. obtusifolium* was one of the fruiting trees studied concerning interactions between fruit flies and parasites [31]. Da Mata et al. [49] studied teenagers ecological knowledge of plants to examine the theoretical and practical implications of ethnobiology, including quixabeira. 

In 2024, two studies were published on the tree and seed of *S. obtusifolium*. Silva et al [52] indicated that quixabeira is one of the plant species with potential economic importance and inclusion in the diet, but highlighted the need to conduct sensory analysis studies. In another investigation by Silva et al. [53], the seeds were subjected to disinfestation treatment using different chemical products (carbendazim, chlorine dioxide, and sodium hypochlorite) with varying times to determine the most efficient agent. In addition, the authors highlighted the use of activated blockade as an *in vitro* culture medium that promotes greater seed germination and potential for seedling propagation. 

Two recent studies (2025) were conducted on quixabeira. *S. obtusifolium* was one of 134 species examined for the floristic composition of an inselberg in the Paraíba region (Brazil) [54]. Other factors examined in the same study were similarity, growth forms and phytogeographic distribution of the species. *S. obtusifolium* was one of the most cited plants in the study by Gonçalves et al. [55] used in the treatment of veterinary diseases, especially wounds, genital diseases and inflammations. The preservation of traditional knowledge and the search for innovations in veterinary health were described by the authors as motivations for conducting more research on the use of plants in ethnoveterinary medicine.

## Conclusions and Future Perspectives

Studies have shown that each part of the tree plays a distinct role: extracts using the leaves are the focus of most phytochemical and biological investigations; the seeds are more related to microbiological activity, including fungicidal and bactericidal effects; and the fruits are investigated for their morphological and phenological aspects, although their antioxidant activity and the presence of bioactive compounds are still underexplored. The primary metabolites found in the different parts of *S. obtusifolium* were phenolic compounds, especially in the leaves, where saponins, alkaloids, glycolipids, and, most notably, the compound NMP, associated with anti-inflammatory, antioxidant, healing, antinociceptive, and neuroprotective actions, were also identified. In addition, terpenoids were also found in the bark and carotenoids in the fruit. The benefits of using *S. obtusifolium*, as reported in the literature, are primarily related to its pharmacological applications, with an emphasis on the species potential in the production of phytotherapeutic products, nutraceuticals, and natural pigments for medicinal use. 

Although the leaves and bark show interesting potential, further studies are still needed on other parts, such as the fruits, in addition to conducting complementary investigations*in vivo* and clinical trials for the proper evaluation of the pharmacological potential of specific compounds. Despite the available literature on*S. obtusifolium*, information regarding its chemical composition and application potential has not yet been fully elucidated. This need for investigation is relevant given the multiple uses of the different parts of the tree and the importance of conserving the species. Future research should prioritize the chemical characterization of the less-studied parts (flowers and fruits), elucidation of the mechanisms of action of the identified compounds, and their potential applications. In addition, research focused on conservation, propagation, and germination is essential to ensure the sustainable use and preservation of *S. obtusifolium*, given its risk of extinction.

## Supplementary Information

Below is the link to the electronic supplementary material.

Supplementary Material 1 (DOCX 349 KB)

## Data Availability

No datasets were generated or analysed during the current study.

## Abbreviations

DTA: Differential thermal analysis
GAT1: Gamma-aminobutyric acid (GABA) transporter
Iba−1: Ionized calcium-binding adapter protein 1
GFAP: Glial fibrillary acidic protein
INPI: National Institute of Industrial Property
LNCaP: Androgen dependent prostate cancer cell line
NMP: N-methyl-(2 S,4R)-trans−4-hydroxy-L-proline
PYR-GC/MS: Pyrolysis coupled to gas chromatography/mass spectrometry
ROS: Reactive Oxygen Species
SEM: Scanning electron microscopy
TG: Thermogravimetry
UFP: Unconventional Food Plants
VDAC−1: Selective channel 1 for stress-dependent anions
