# Supplementary material for: Sideroxylon Obtusifolium (Roem. & Schult.) TD Penn: Knowledge and Potentialities
Source: Plant Foods Hum Nutr. 2026 Jun 22;81(3):81. doi: 10.1007/s11130-026-01475-3 (PMC13287164; doi:10.1007/s11130-026-01475-3)
Supplement: Supplementary file 1 — Supplementary Material 1 (DOCX 349 KB) [file 11130_2026_1475_MOESM1_ESM.docx]

***Sideroxylon obtusifolium* (Roem. & Schult.) TD Penn: Knowledge and potentialities**

**Luísa dos Santos Conceição^1^; Sanket Prakash Vanare^2^; Ronald Bruce Pegg ^2^; Deborah Murowaniecki Otero^1,3^**

^¹^Graduate Program in Food Science, Faculty of Pharmacy, Federal University of Bahia, Campus Ondina, Salvador, Bahia, 40170115, Brazil.

^2^Department of Food Science & Technology, University of Georgia, Athens, Georgia 30602, United States of America.

^3^Graduate Program in Food, Nutrition, and Health, Nutrition School, Federal University of Bahia, Campus Canela, Salvador, Bahia, 40110907, Brazil. https://orcid.org/0000-0001-9792-3992

Corresponding author: [deborah.otero@ufba.br](mailto:deborah.otero@ufba.br)

**2. Procedure and Data Analysis**

The electronic databases selected for this study were SciELO, Springer Link, PubMed, and ScienceDirect. A literature search was conducted in May 2025, considering all titles or abstracts of studies that contained the descriptor terms *Sideroxylon obtusifolium* (scientific name) and quixabeira/quixaba (popular names).

The inclusion criteria included articles published in the last fifteen years, related to the descriptor terms: bioactive compounds, antioxidant activity, biological properties, proximal, nutritional, chemical, and physicochemical composition of *S. obtusifolium*. Exclusion criteria were duplicate studies and articles outside the scope of the study. In addition, searches in ESPACENET and the National Institute of Industrial Property (INPI) did not yield any results on patents related to quixaba. In total, 35 articles were included for this review (Figure S1).


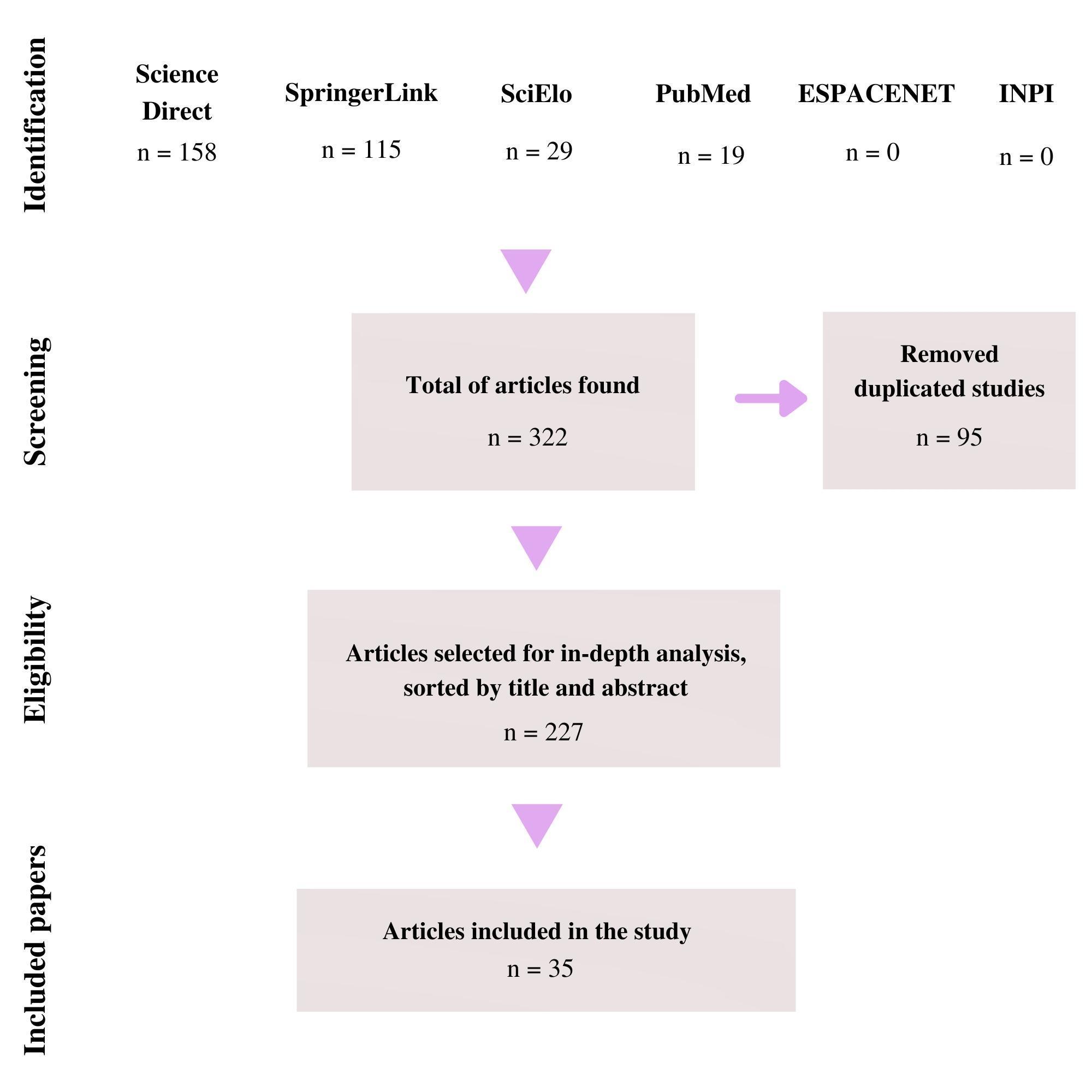


**Fig. S1** PRISMA flow diagram. The figure illustrates each step of the search strategy performed.

The chosen time interval offers a critical and integrated view of the state of the art, facilitating the identification of trends, consolidation of research lines, and the evolution of the analytical methods employed. Furthermore, the time delimitation is essential for identifying trends, knowledge gaps, convergences, and divergences among the reported results, as well as for evaluating the robustness of the available evidence.

**Results and discussion**

**Table S1.** Extraction of compounds present in different parts of *S. obtusifolium*

| **Part of the tree** | **Solvent** | **Extraction method** | **Compound identified** | **Biological action** | **Reference** |
| --- | --- | --- | --- | --- | --- |
| Leaves | Methanol | Extracted with Soxhlet | N-methyl-(2S,4R)-trans-4-hydroxy-l-proline (NMP) | Antinociceptive and anti-inflammatory | [8] |
| Leaves | Ethanol | Maceration with solvent | Quercetin-3- rhamnosyl-(1-6)-galactoside,  Hyperoside | Antiparasitic | [9] |
| Bark | ethyl acetate | - | Epicatechin,  Heptahydroxyflavan,  Proanthocyanidins | Antiandrogenic, antioxidant, antibacterial | [15] |
| Leaves | Methanol | Immersion in solvent for 72 hours | Total phenols,  Flavonoids,  Pyrogallic tannins,  Flavones,  Flavonols,  Xanthones,  Aurones,  Flavononols,  Leucoanthocyanidins,  Catechins,  Flavonones,  Alkaloids | Anti-inflammatory and antibacterial | [16] |
| Leaves | Hydroalcoholic solution | Maceration with solvent | Flavonoids | Antifungal activity (*C. albicans)* | [17] |
| Leaves | Ethanol | Maceration with solvent | Flavonoids,  Saponins,  Catechin,  Gingerglycolipid A | - | [20] |
| Fruit | Acetone,  Hydrochloric acid and chloroform | Solvent extraction | Anthocyanins | - | [24] |
| Leaves | Methanol | extracted with Soxhlet | N-Methyl-(2S,4R)-trans-4-Hydroxy-L-Proline (NMP) | Antioxidant, anti-inflammatory, and healing | [38] |
| Leaves | Methanol | Immersion in solvent for 72 hours | N-methyl-(2S,4R)-trans-4-hydroxy-L-proline (NMP) | Anticonvulsant, anti-inflammatory, and neuroprotective | [47] |
| Leaves | Methanol | Immersion in solvent for 72 hours | N-methyl-(2S,4R)-trans-4-hydroxy-L-proline (NMP) | Neuroprotection and antioxidant activity | [48] |
| Bark and leaves | Ethanol | Maceration with solvent | Polyphenols,  Flavonoids,  Condensed tannins | Antifungal activity (*C. albicans)* | [49] |
| Inner bark | Ethanol | Maceration with solvent | Total phenols,  Tannins,  Flavonols,  Flavanonols,  Flavanones,  Xanthones,  Steroids,  Triterpenoids,  Saponin heterosides | Antioxidant activity | [50] |

**Table S2.** Articles found for review

| **Title** | **Objective** | **Country of publication** | **Part of Tree** | **Reference** |
| --- | --- | --- | --- | --- |
| Local botanical knowledge of farmers of the semiarid of Paraíba state, Northeast of Brazil | Describe and analyze the interrelationship between residents in the rural community of Santa Rita (Cariri of Paraíba) and useful woody plants, evaluating their availability through ethnobotanical and plant inventory. | Brazil | Tree | [3] |
| The anti-inflammatory effects of N-methyl-(2S,4R)-trans-4-hydroxy-l-proline from *Sideroxylon obtusifolium* are related to its inhibition of TNF-alpha and inflammatory enzymes | Evaluate the *in vitro* antimicrobial activity of extracts and chemical fractions of *S. obtusifolium* on *S. mutan*s, *Streptococcus oralis, Streptococcus salivarius, Streptococcus parasanguinis, and C. albicans*, as well as identify the classes of chemicals in the bioactive extracts with the best activity. | Brazil | Bark and Leaves | [8] |
| Nanoemulsion of *Sideroxylon obtusifolium* as an Alternative to Combat Schistosomiasis | Evaluate the effectiveness of nanoemulsion produced with the extract-SOB of *S. obtusifolium* leaves, demonstrating this activity at three points in the biological cycle of the disease. | BR | Leaves | [9] |
| Phenology of *Sideroxylon obtusifolium* (Roem. & Schult.) T.D.Penn. in a Caatinga area, Boa Vista – Paraíba state | Evaluate the phenological stages of *Sideroxylon*  *obtusifolium* and their relationship with the local environmental conditions. | BR | Tree | [10] |
| A proline derivative-enriched methanol fraction from *Sideroxylon obtusifolium* leaves (MFSOL) stimulates human keratinocyte cells and exerts a healing effect in a burn wound model | Investigate the healing effects of MFSOL on human keratinocyte cells (HaCaT) and experimental burn model injuries. | Brazil | Leaves | [11] |
| Contributions of Annual Phenological Intensity to the Production of Tannins in *Sideroxylon obtusifolium* in Brazilian Semi-arid | Assess the relationship between phenological intensity and tannin production in *Sideroxylon obtusifolium* (Roem. & Schult.) T.D.Penn. | Brazil | Bark  and inner bark | [12] |
| Reproductive biology of *Sideroxylon obtusifolium* (Roem. & Schult.) T.D. Penn. (Sapotaceae) in the semiarid region of Bahia | Study phenology, reproductive biology and flower visitors of *Sideroxylon obtusifolium* in caatinga area. | Brazil | Tree | [13] |
| Multiple readout assay for hormonal (androgenic and antiandrogenic) and cytotoxic activity of plant and fungal extracts based on differential prostate cancer cell line behavior | Detect influences of plant or fungal extracts and derived fractions on androgen receptor signaling pathways, a differentiating cell proliferation assay was established, which enables the simultaneous detection of hormonal and cytotoxic effects | Germany | Bark | [15] |
| Evaluation of the Topical Anti-inflammatory Activity and Antibacterial Activity of Methanol Extract in the *Sideroxylon obtusifolium* Leaves | Investigate the topical antibacterial and anti-inflammatory activity of the methanol extract obtained from the leaves of *S. obtusifolium*. | Brazil | Leaves | [16] |
| Antifungal potential and biosafety of native plants from the Brazilian Restinga ecosystem | Evaluate the fungicidal potential through *in vitro* antimicrobial assays of *Sideroxylon obtusifolium* and Annona acutiflora leaf extracts | Brazil | Leaves | [18] |
| Extracts of Caesalpinia ferrea and Trichoderma sp. on the control of Colletotrichum sp. transmission in *Sideroxylon obtusifolium* seeds | Evaluate the effect of biotech treatments in controlling the transmission of *Colletotrichum sp*. in seeds of *S. obtusifolium*. | Brazil | Seeds and seedlings | [19] |
| Metabolite profiling of the leaves of the Brazilian folk medicine *Sideroxylon obtusifolium* | Investigation of metabolite profiling of the leaves of *S. obtusifolium.* | Switzerland | Fruit | [20] |
| Seed maturation of *Sideroxylon obtusifolium* [(Roem. & Schult.) T.D. Penn.] at different times of collecting | Study the maturation process of *Sideroxylon obtusifolium* seeds, aiming to determine the physiological maturity point. | BR | Seed | [22] |
| Morphological characterization of fruits, seeds and germination of *Sideroxylon obtusifolium* (Roem. E Schult.) Penn. (Sapotaceae) | Characterize morphologically internal and external structures of fruits and seeds of *Sideroxylon obtusifolium* (Roem. e Schult.) Penn. and to describe and illustrate the external  Morphology. | Brazil | Fruit and seed | [23] |
| Antioxidant activity of anthocyanins from quixabeira (*Sideroxylon obtusifolium*) fruits | Research was to evaluate the antioxidant activity of anthocyanins from mature fruits which were obtained at a semi-arid region of Paraiba/BR. | Brazil | Fruit | [24] |
| Fruiting phenology and consumption of zoochoric fruits by wild vertebrates in a seasonally dry tropical forest in the Brazilian Caatinga | Describe and evaluate the fruit phenology of zoochoric plants and the use of this resource by wild vertebrates in a seasonally dry tropical forest in the Furna Feia National Park, an area of Brazilian Caatinga | Brazil | Seeds | [28] |
| Methods for overcoming dormancy of quixabeira seeds (*Sideroxylon obtusifolium* (Roem.&Schult.) T.D.Penn.) | Determine the most efficient method to overcome the seed coat dormancy in seeds of *Sideroxylon obtusifolium* (Roem. & Schult.) T.D.Penn. | Brazil | Fruit and seed | [30] |
| Fruit Flies (Diptera: Tephritoidea) and Parasitoids (Hymenoptera) Associated with Native Fruit Trees in the Chaco Biome | Evaluate the tritrophic interactions of fruit fies and their parasitoids occurring in native fruits in the Chaco Biome. | BR | Fruit | [33] |
| Reproductive phenology in the standardization of tannins in plant drugs of specimens of *Sideroxylon obtusifolium* (Roem. & Schult.) T.D. Penn | Develop and validate an analytical model for the selection of determinant parameters in the collection and sustainable medicinal use of the vegetable drug of *Sideroxylon obtusifolium* (Roem. & Schult.) T.D. Penn. | Brazil | Caule | [32] |
| The Wound Healing Property of N-Methyl-(2S,4R)-trans-4-Hydroxy-L-Proline from *Sideroxylon obtusifolium* is Related to its Anti-Inflammatory and Antioxidant Actions | Investigate the topical effects of the NMP gel on a mice wound-induced model | Brazil | Leaves | [36] |
| Chemical characterization of native wild plants of dry seasonal forests of the semi-arid region of northeastern Brazil | Determine the centesimal profile and quantify the total amount of anthocyanins, flavonols and carotenoids of native species of the Caatinga. | Brazil | Fruit | [38] |
| A Proline Derivative-Enriched Fraction from *Sideroxylon obtusifolium* Protects the Hippocampus from Intracerebroventricular Pilocarpine-Induced Injury Associated with Status Epilepticus in Mice | Study the effects of NMP on the behavioral and brain changes occurring after intracerebroventricular (icv) pilocarpine (Pilo)-induced status epilepticus (SE) and brain damage, by considering the expression of GAT1, neuroinflammation, and gliosis, as evaluated by Nissl staining, immunohistochemistry assays, and western blotting. | BR | Leaves | [45] |
| N-Methyl-(2S,4R)-trans-4-hydroxy-L-proline, the major bioactive compound from *Sideroxylon obtusifolium*, attenuates pilocarpine-induced injury in cultured astrocytes | Investigate the effect of N-methyl-(2S,4R)-trans-4-hydroxy-L-proline (NMP) on astrocytes exposed to cytotoxic concentrations of pilocarpine. | BR | Leaves | [46] |
| Evaluation of the cicatrizing, topical anti-inflammatory and antioxidant activities of the ethanol extract of *Sideroxylon obtusifolium* | Evaluate the effect of the ethanol extract (EE) of *S. obtusifolium* on the cicatrization and local anti-inflammatory  activities using a model of wound healing in rats. | Brazil | Inner bark | [47] |
| Antimicrobial Potential of Plant Extracts and Chemical Fractions of *Sideroxylon obtusifolium* (Roem. & Schult.) T.D. Penn on Oral Microorganisms | Chemical characterization and exploration of species from the Caatinga biome in Brazil, *Sideroxylon obtusifolium* T.D. Penn (Sapotacea) and *Syzygium cumini* (L.) Skeels (Myrtaceae), regarding their antifungal potential against C. albicans. | Brazil | Leaves | [48] |
| Conservation priorities for medicinal woody species in a cerrado area in the Chapada do Araripe, northeastern Brazil | Classify medicinal woody species for priority conservation site in an area of cerrado disjoint in Chapada do Araripe, northeastern Brazil. | Brazil | Bark and Leaves | [51] |
| Teenagers’ ecological knowledge about dry forests in Northeastern Brazil: theoretical and practical implications in ethnobiology | Examine adolescents' knowledge and perception of forests and how this knowledge is patterned across different socioeconomic variables. | BR | Tree | [52] |
| *Sideroxylon obtusifolium* herbal medicine characterization using pyrolysis GC/MS, SEM and different thermoanalytical techniques | Characterize by analytical techniques *Sideroxylon obtusifolium* herbal medicine raw material derived from the leaves of the plant in different particle sizes, to obtain information which assist the popular use dissemination of natural products for medicinal purposes. | Brazil | Leaves | [53] |
| Potential of quixaba (*Sideroxylon obtusifolium*) latex as a milk-clotting agent | Evaluate the potential of latex from *Sideroxylon obtusifolium* as a source of milk-clotting proteases and to partially characterize the enzyme. | Brazil | Stems | [54] |
| Biodiverse food plants in the semiarid region of Brazil have unknown potential: A systematic review | Identify biodiverse food plants occurring in the Caatinga biome, Brazil, strategic for the promotion of food and nutrition security. | Brazil | Fruit | [55] |
| Climate change may alter the availability of wild food plants in the Brazilian semiarid | Estimate the future availability of nutritionally and economically important Wild food plants (WFP) species in the Brazilian semiarid and determine their spatiotemporal variation in future scenarios of climate change. | Brazil | Tree | [56] |
| *In vitro* establishment of *Sideroxylon obtusifolium* (Roem. & Schult.) T. D. Penn | Determine the most efficient chemical agent for seed disinfestation and the culture medium for *in vitro* establishment of this species. | Brazil | Seeds | [57] |
| Are these islands isolated? Floristic diversity in inselberg habitats of the Caatinga, Northeastern Brazil | Examined the floristic composition of inselbergs in the Caatinga, Northeastern Brazil, and the associations among species across different habitats. | Brazil | Tree | [58] |
| Use and utility redundancy of medicinal  plants in ethnoveterinary medicine by local  populations of the Brazilian Caatinga | Document Caatinga plants known for treating diseases in ethnoveterinary medicine; and evaluate the utility redundancy of veterinary diseases that affects domestic animals indicated by local populations of a region of the state of Paraíba | Brazil | Tree | [59] |


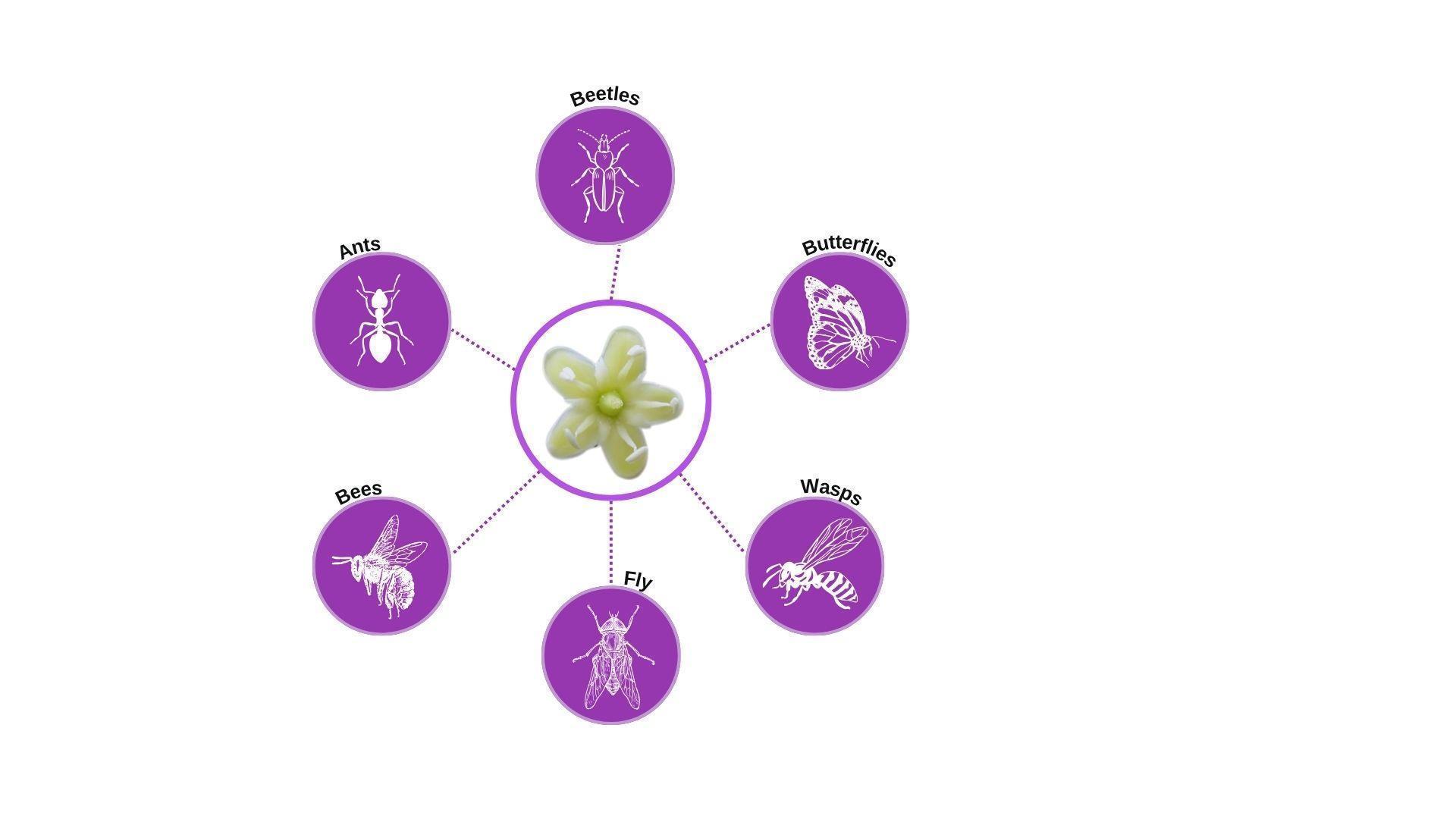


**Fig. S2**  Floral visitors identified in the quixabeira (figure was created with canva.com).


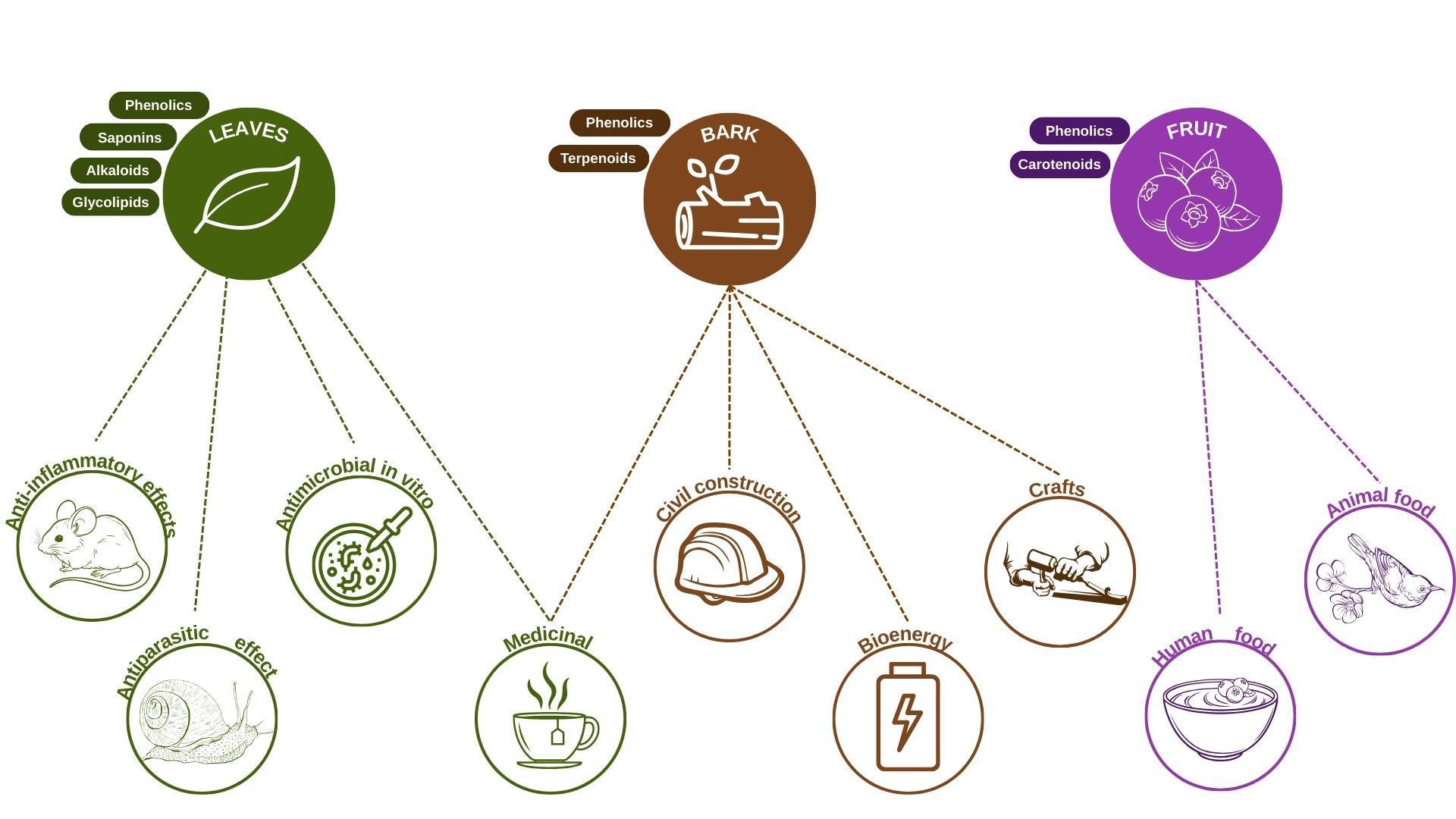


**Fig. S3** Uses of different parts of *S. obtusifolium* and distribution of associated chemical compounds (figure was created with canva.com).


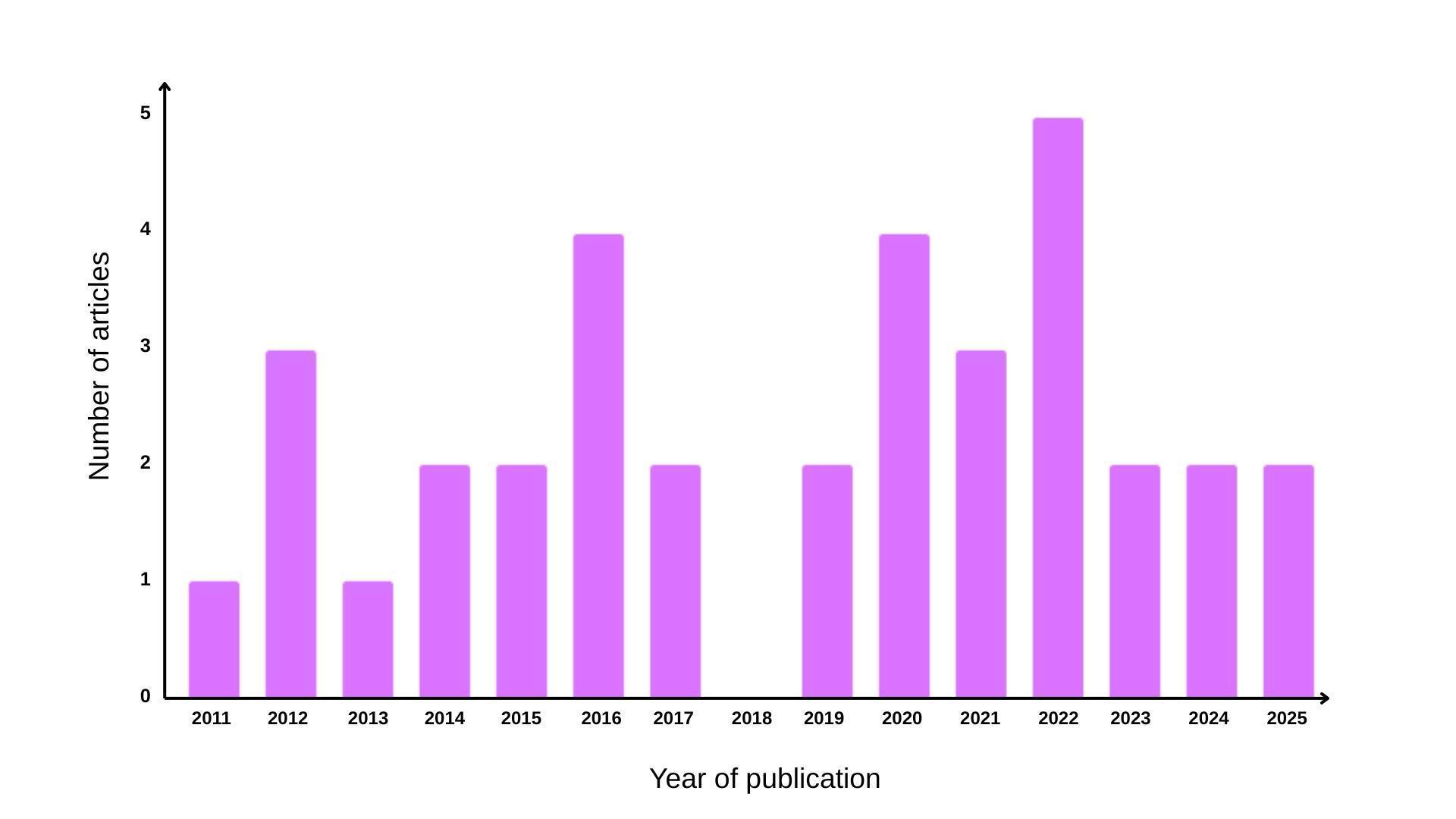


**Fig. S4** Temporal evolution of articles found in the literature (figure was created with Excel).

**References**

1. Harris J, van Zonneveld M, Achigan-Dako EG et al (2022) Fruit and vegetable biodiversity for nutritionally diverse diets: Challenges, opportunities, and knowledge gaps. Glob Food Sec 33:100618. https://doi.org/10.1016/j.gfs.2022.100618
2. Barbosa EU, Carvalho TK, Ferreira EC, Santos SS, Lucena RF (2020) Local botanical knowledge of farmers of the semiarid of Paraíba State, Northeast of Brazil. Polibotánica 50:191-208. https://doi.org/10.18387/polibotanica.50.13
3. Cardoso PD, da Silva IN, Ferreira-Ribeiro CD, Murowaniecki OD (2023) Nutritional and technological potential of cactus fruits for insertion in human food. Crit Rev Food Sci Nut 63:4053-4069. https://doi.org/10.1080/10408398.2021.1997906
4. Liberato P, Lima T, Silva B (2019) UFPs - Unconventional food plants and their nutritional benefits. Environmental smoke 2:102-111. https://doi.org/10.32435/envsmoke.201922102-111
5. Costa L, Trindade P, da Silva Cardoso P, Colauto B, Linde A, Otero D (2023) *Pachira aquatica* (Malvaceae): An unconventional food plant with food, technological, and nutritional potential to be explored. Food Res Int 164:112354. https://doi.org/10.1016/j.foodres.2022.112354
6. Bezerra JA, Brito MD (2020) Nutritional and antioxidant potential of unconventional food plants and their use in food: Review. Research, Society and Development 9:e369997159. <https://doi.org/10.33448/rsd-v9i9.7159>
7. Aquino PE, Magalhães TR, Nicolau LA, et al (2017) The anti-inflammatory effects of N-methyl-(2S,4R)-*trans*-4-hydroxy-L-proline from *Syderoxylon obtusifolium* are related to its inhibition of TNF-alpha and inflammatory enzymes. Phytomed 24:14-23. https://doi.org/10.1016/j.phymed.2016.11.010
8. Rangel LD, de Oliveira AP, Falcão DQ, et al (2022) Nanoemulsion of *Sideroxylon obtusifolium* as an alternative to combat Schistosomiasis. Front Plant Sci 13:853002. https://doi.org/10.3389/fpls.2022.853002
9. Cruz FR, Silva RD, Alves EU, Rodrigues CM (2022) Phenology of *Sideroxylon obtusifolium* (Roem. & Schult.) T.D. Penn. in a Caatinga area, Boa Vista – Paraíba state. Ci Fl 32:653-672. <https://doi.org/10.5902/1980509844038>
10. Souza TF, Pierdoná TM, Macedo FS, et al (2021) A proline derivative-enriched methanol fraction from *Sideroxylon obtusifolium* leaves (MFSOL) stimulates human keratinocyte cells and exerts a healing effect in a burn wound model. Braz J Med Biol 54:e10700. https://doi.org/10.1590/1414-431X2021e10700
11. Gomes AC, Andrade FH, Lacerda AV, Macêdo RO (2021) Contributions of annual phenological intensity to the production of tannins in *Sideroxylon obtusifolium* in Brazilian semiarid. Floresta e Ambient 28:e20210027. https://doi.org/10.1590/2179-8087-FLORAM-2021-0027
12. Kiill LH, Martins CT, Silva PP (2014) Reproductive biology of *Sideroxylon obtusifolium* (Roem. & Schult.) T.D. Penn. (Sapotaceae) in the semiarid region of Bahia. Rév Árvore 38:1015-1025. https://doi.org/10.1590/S0100-67622014000600006
13. Coradin L, Camillo J, Pareyn F (2018) Espécies nativas da flora brasileira de valor econômico atual ou potencial: plantas para o futuro: região Nordeste. Brasília (DF): MMA – Ministério do Meio Ambiente. https://www.alice.cnptia.embrapa.br/alice/bitstream/doc/1102927/1/LivroNordeste12018.pdf. Accessed 28 July 2025.
14. Bobach C, Schurwanz J, Franke K et al (2014) Multiple readout assay for hormonal (androgenic and antiandrogenic) and cytotoxic activity of plant and fungal extracts based on differential prostate cancer cell line behavior. J Ethnopharmacol, 155:721-730. https://doi.org/10.1016/j.jep.2014.06.008
15. Aquino P, Gomes F, Pereira N, Nascimento E, et al (2016) Evaluation of the topical anti-inflammatory activity and antibacterial activity of methanol extract in the *Sideroxylon obtusifolium* leaves. Acta Biol. Colomb. 21:131-140. https://doi.org/10.15446/abc.v21n1.48170
16. Pereira JV, Freires IA, Castilho AR, et al (2016) Antifungal potential of *Sideroxylon obtusifolium* and *Syzygium cumini* and their mode of action against *Candida albicans*. Pharm Biol 54:2312-2319. https://doi.org/10.3109/13880209.2016.1155629
17. Duarte JA, Fiaux SB, Barbosa E, et al (2022). Antifungal potential and biosafety of native plants from the Brazilian Restinga ecosystem. Clean Eng Technol 8:100493. https://doi.org/10.1016/j.clet.2022.100493
18. Melo PA, Alves EU, Martins CC, et al (2016) Extracts of *Caesalpinia ferrea* and Trichoderma sp. on the control of *Colletotrichum* sp. transmission in *Sideroxylon obtusifolium* seeds. Rev Bras Plantas Med. 18:494–501. https://doi.org/10.1590/1983-084X/15_191
19. Oliveira AP, Raith M, Kuster RM, et al (2012). Metabolite profiling of the leaves of the Brazilian folk medicine *Sideroxylon obtusifolium*. Planta Med, https://doi.org/10.1055/s-0031-1298269
20. Lorenzi H (1998) Brazilian trees: manual for identification and cultivation of tree plants native to Brazil, 2nd edn. Plantarum, Nova Odessa, pp 323
21. Sena DV, Alves EU, Araújo LR, et al (2022) Seed maturation of *Sideroxylon obtusifolium* [(Roem. & Schult.) TD Penn.] at different times of collecting. Ci Fl 32:1106-1124. https://doi.org/10.5902/1980509834217
22. Silva KB, Alves EU, Bruno RD, et al (2012) Morphological characterization of fruits, seeds, and germination of *Sideroxylon obtusifolium* (Roem. and Schult.) Penn. (Sapotaceae). Árvore 36:59-64. https://doi.org/10.1590/S0100-67622012000100007
23. Figueiredo FJ and Lima VL (2015) Antioxidant activity of anthocyanins from quixabeira (*Sideroxylon obtusifolium*) fruits. Rev Bras Plantas Med 17:473-479. https://doi.org/10.1590/1983-084X/14_005
24. Barbosa DA (2018) Ethnobotany and phytochemical screening of *Sideroxylon obtusifolium* (Roem. & Schult.) T.D. Penn. (Quixabeira), Cabaceiras, Semiárido da Paraíba. Master's thesis, Federal University of Paraíba
25. NEMA: Center for Ecology and Environmental Monitoring (2021) https://nema.univasf.edu.br/index.php?page=newspaper&record_id=81. Accessed 28 July 2025
26. Silva EE, Paixão VH, Torquato JL, Lunardi DG, Lunardi VL (2020) Fruiting phenology and consumption of zoochoric fruits by wild vertebrates in a seasonally dry tropical forest in the Brazilian Caatinga. Acta Oecol 105:103553. https://doi.org/10.1016/j.actao.2020.103553
27. Silva FF, Dantas BF (2017) *Sideroxylon obtusifolium* (Humb. ex Roem. & Schult.) TD Penn. Quixabeira. Embrapa Semiárido-Nota Técnica/Nota Científica (ALICE). http://www.alice.cnptia.embrapa.br/alice/handle/doc/1074013. Accessed 27 July 2025
28. Rebouças AC, Matos VP, Ferreira RL, et al (2012) Methods for overcoming dormancy of quixabeira seeds (*Sideroxylon obtusifolium* (Roem. & Schult.) TD Penn.). Ci Fl 22:183-192. https://doi.org/10.5902/198050985090
29. Gomes R, Pinheiro MC, Lima HA, Santiago-Fernandes LD (2010). Floral biology of *Manilkara subsericea* and *Sideroxylon obtusifolium* (Sapotaceae) in Restinga. Braz J Bot 33:271-283. https://doi.org/10.1590/S0100-84042010000200008
30. Gomes AC, de Andrade FH, de Lacerda AV, Macêdo RO (2021) Reproductive phenology in the standardization of tannins in plant drugs of specimens of *Sideroxylon obtusifolium* (Roem. & Schult.) TD Penn. Braz J Bot 44:561-573. https://doi.org/10.1007/s40415-021-00726-8
31. Coelho JB and Uchoa MA (2023) Fruit flies (Diptera: Tephritoidea) and parasitoids (Hymenoptera) associated with native fruit trees in the Chaco biome. Neotrop Entomol 52:629-641. https://doi.org/10.1007/s13744-023-01055-z
32. Correia LP, Santana CP, Medeiros AC, Macêdo RO (2016) *Sideroxylon obtusifolium* herbal medicine characterization using pyrolysis GC/MS, SEM and different thermoanalytical techniques. J Therm Anal Calorim 123:993-1001. https://doi.org/10.1007/s10973-015-4986-1
33. Vizzotto, M (2012) Propriedades funcionais de pequenas frutas. https://www.alice.cnptia.embrapa.br/alice/bitstream/doc/939258/1/MarciaVizzottop8488.pdf. Accessed 23 July 2025
34. Milanezzi GC (2022) Bioactive compounds in Brazilian exotic fruits: a literature review. Braz J Dev 8:52376-52385. https://doi.org/10.34117/bjdv8n7-246
35. Aquino PE, de Souza TD, Santos FA, et al (2019) The wound healing property of N-Methyl-(2S, 4 R)-*trans*-4-hydroxy-L-proline from *Sideroxylon obtusifolium* is related to its anti-inflammatory and antioxidant actions. J Evid Based Integr Med 24:2515690X19865166. https://doi.org/10.1177/2515690X19865166
36. do Nascimento VT, De Moura NP, da Silva Vasconcelos MA, et al (2011) Chemical characterization of native wild plants of dry seasonal forests of the semiarid region of northeastern Brazil. Food Res Int 44:2112-2119. https://doi.org/10.1016/j.foodres.2010.12.024
37. Rocha WS, Lopes RM, Silva DB, et al (2011) Total phenolics and condensed tannins in native fruits from Brazilian savanna. Rev Bras Frutic 33:1215-1221. https://doi.org/10.1590/S0100-29452011000400021
38. Rossi IS, Costa JB, Nascimento LG, de Carvalho AF (2022) Stability of açaí anthocyanins: a brief review. J Eng Exact Sci 8:14880-01a. https://doi.org/10.1590/0100-29452017564
39. Abe LT, Mota RV, Lajolo FM, Genovese MI (2007) Phenolic compounds and antioxidant activity of *Vitis labrusca* and *Vitis vinifera* cultivars. Food Sci Technol 27:394-400. [https://doi.org/10.1590/S0101-2061200700020003](https://doi.org/10.1590/S0101-20612007000200032)2
40. Silva AK, Beckman JC, da Cruz Rodrigues AM, da Silva LH (2017) Nutritional composition and antioxidant capacity of açaí pulp (*Euterpe oleracea* M.). Rev Bras Tecnol Agroind 11. https://doi.org/10.3895/rbta.v11n1.2829
41. Singh T, Pandey VK, Dash KK, et al (2023) Natural bio-colorant and pigments: Sources and applications in food processing. J Agric Food Res 12:100628. https://doi.org/10.1016/j.jafr.2023.100628
42. Koop BL, da Silva MN, da Silva FD, et al (2022) Flavonoids, anthocyanins, betalains, curcumin, and carotenoids: Sources, classification, and enhanced stabilization by encapsulation and adsorption. Food Res Int 153:110929. https://doi.org/10.1016/j.foodres.2021.110929
43. Aquino PE, Rabelo BJ, de Souza NT, et al. (2020) A proline derivative-enriched fraction from *Sideroxylon obtusifolium* protects the hippocampus from intracerebroventricular pilocarpine-induced injury associated with status epilepticus in mice. Int J Mol Sci 21:4188. https://doi.org/10.3390/ijms21114188
44. Aquino PE, de Siqueira EA, Paes LC, et al (2022) N-Methyl-(2S, 4R)-*trans*-4-hydroxy-L-proline, the major bioactive compound from *Sideroxylon obtusifolium*, attenuates pilocarpine-induced injury in cultured astrocytes. Braz J Med and Biol Res 55. https://doi.org/10.1590/1414-431X2022e12381
45. Sampaio TP, Cartaxo-Furtado NA, de Medeiros AC, et al (2017) Antimicrobial potential of plant extracts and chemical fractions of *Sideroxylon obtusifolium* (Roem. & Schult.) TD Penn on oral microorganisms. J Contemp Dent Pract 18:392-398. https://doi.org/10.5005/jp-journals-10024-2053
46. Leite NS, Lima AP, Araújo N, Estevam CS, et al (2015) Evaluation of the cicatrizing, topical anti-inflammatory and antioxidant activities of the ethanol extract of *Sideroxylon obtusifolium*. Rev Bras Plantas Med 17:164-170. https://doi.org/10.1590/1983-084X/09_189
47. Raza A, Xu X, Sun H, et al (2017). Pharmacological activities and pharmacokinetic study of hyperoside: A short review. Tropical Journal of Pharmaceutical Research 16:483-489. https://doi.org/10.4314/tjpr.v16i2.30
48. Ribeiro DA, Macedo DG, de Oliveira LG, et al (2019) Conservation priorities for medicinal woody species in a cerrado area in the Chapada do Araripe, Northeastern Brazil. Environ Dev Sustain 21:61-77. https://doi.org/10.1007/s10668-017-0023-9
49. Da Mata PT, de Oliveira AR, Arnan X, et al (2023) Teenagers' ecological knowledge about dry forests in Northeastern Brazil: theoretical and practical implications in ethnobiology. SN SS, 3:58. https://doi.org/10.1007/s43545-023-00636-4
50. Silva AC, Nascimento TC, Silva AS, et al (2013) Potential of quixaba (*Sideroxylon obtusifolium*) latex as a milk-clotting agent. Food Sci Technol 33:494-499. https://doi.org/10.1590/S0101-20612013005000075
51. Jacob MC, Araujo de Medeiros MF, Albuquerque UP (2020) Biodiverse food plants in the semiarid region of Brazil have unknown potential: A systematic review. PLoS One 15:e0230936. https://doi.org/10.1371/journal.pone.0230936
52. Silva ASS, Arnan X, Medeiros PM (2024) Climate change may alter the availability of wild food plants in the Brazilian semiarid. Reg Environ Change 24:86. https://doi.org/10.1007/s10113-024-02250-3
53. Silva IT, Lima APPS, Santana JRF (2024). *In vitro* establishment of *Sideroxylon obtusifolium* (Roem. & Schult.) T. D. Penn. Rev Caatinga 37:e11850. https://doi.org/10.1590/1983-21252024v3711850rc
54. Cordeiro JMP, Souza BID, Souza AFD, et al (2025) Are these islands isolated? Floristic diversity in inselberg habitats of the Caatinga, Northeastern Brazil. Biodiversity and conservation 34:2359-2379. https://doi.org/10.1007/s10531-025-03065-2
55. Gonçalves, JRF, Pedrosa KM, Ramos MB et al (2025) Use and utility redundancy of medicinal plants in ethnoveterinary medicine by local populations of the Brazilian Caatinga. J Ethnobiology Ethnomedicine 21:22. https://doi.org/10.1186/s13002-025-00762-8
